# Patient Process-Based Well-Being With the Support of a Close Person

**DOI:** 10.1177/23743735231155802

**Published:** 2023-02-12

**Authors:** Sanna Pauliina Ryynänen

**Affiliations:** 1 410680 Faculty of Social Sciences, University of Lapland, Rovaniemi, Finland; 2 Administration, 159841 Helsinki University Central Hospital, Helsinki, Finland

**Keywords:** quality of life, clinician–patient relationship, relationships in healthcare, patient engagement, communication, healthcare planning or policy, patient/relationship-centered skills, organizational culture

## Abstract

The area of patient well-being, from medical care to everyday life, can be seen as a mutual, value-driven co-creative whole. However, the principle of customer-centricity has not sufficiently taken into account the patient's need for a close person's support in the care and home environments, especially in Nordic healthcare systems. The patient's well-being in healthcare can be viewed as a process-like service experience, including perceptions of their own well-being and a need for support in confronting the deterioration in their health. Therefore, well-being in the care process is not based solely on treatment results. Patient care proceeds as a service process in which mutual value is formed through the exchange of information and mutual understanding between a patient (ie, consent provider) within their social context (ie, support provider) and a service provider (ie, healthcare professional) in achieving care results. In a professional and organization-oriented care culture, the support of a close person can be seen as an expansion of the value network of patient care, which, in addition to providing individual and organizational human resource benefits, improves the service process.

## Introduction

In healthcare systems, the emphasis is often on customer-centricity or customer orientation. For quite a long time, the patient has been at the center of the provision and development of care, at least on a principled level, but due to this emphasis on patient-centeredness, the role of close persons, such as family members, relatives, or friends, has been almost nonexistent, especially when it comes to the care of suddenly ill and previously healthy adult patients with legal capacity. In the midst of symptoms and illness, the patient's functional capacity and, consequently, their ability to attend to not only their physical condition but also daily living and personal matters are often impaired. They may need the help of a close person in many daily activities and during the care process as in- or outpatients. Traditionally, Nordic healthcare systems have not relied as heavily on the role of family members or other close persons as have healthcare systems in southern and eastern Europe (1,2).

In Finnish healthcare, the close person's role mainly involves acting as the patient's contact person during the care process. At the beginning of the care relationship, the patient chooses a contact person who acts, as the term indicates, as an information broker to others to whom the patient has given consent to receive information (Act on the Status and Rights of Patients 785/1992). This is the close person's main task, and much depends on the operating principles of the involved specialty unit and especially, the doctor's acceptance of the close person's role. The term close person is widely used in Finnish healthcare and legislation, such as the aforementioned Act, and it refers to any person that the patient thinks is close to them.

The role of a close person in patient care could be better considered, even more so due to the lack of human resources in Finnish healthcare organizations. A shortage of nurses has clearly contributed to the difficulty of implementing care processes (treatment during the care period) and care paths (the flow of care from primary healthcare to specialized medical care and from there to a relevant facility or home), as well as to much longer wait times for care due to the Covid-19 pandemic (ie, a backlog of care). This shortage of nurses, a global phenomenon for which there are many reasons, has given rise to difficult situations for patients, such as delayed treatments, and has affected the entire healthcare system (3,4).

### Key Factors

The patient, who is at the center of the care process, is the person most strongly impacted by its benefits and harms. While patient outcomes are linked to the implementation of care and the allocation of human resources, few ideas for solving the nursing shortage have been proposed or studied. One recent proposal to help relieve this problem is the use of care assistants in hospitals. However, despite their training, care assistants are not health professionals; they merely support nurses in assisting with patients’ daily activities. In practice, they do the same tasks as a close person would in the patient's home environment.

The following scenario is an example of the role a close person plays. An adult patient arrives at the emergency room, escorted by a close friend, late at night. She has a shard of glass in her eye from an accident at work. At the entrance to the emergency room, the close friend is turned away. This is done systematically for everyone who is not a patient's next of kin (there is a note with the information on the emergency room entrance). In the waiting room, the patient sits for 5 h, during which time nothing happens. The patient's eye symptoms are painful, and the risk of eye damage makes her fearful. Even so, no staff member will inform her about how long she might have to wait, and there is no notice board with wait times. At its worst, a patient might wait for more than 10 h to receive care in the emergency room. Patients in need of urgent care are treated, but others are forced to wait for long periods of time. After 5 h, the patient calls her friend, who suggests a private occupational healthcare service arranged by the employer, where a patient can get an ophthalmologist's appointment within 2 h. The patient's close person is allowed inside the ophthalmologist's waiting room. She waits until the patient's treatment is complete. On the way home, they go together to the local pharmacy to get the prescribed medicine. The patient still has difficult keeping her injured eye open due to swelling. However, the patient is satisfied and feels good after receiving treatment and the support of a close person.

In addition to being denied the ability to accompany patients during treatment, close persons have complained about not receiving adequate information during the care process. For example, when a patient is transferred from the emergency room to the ward, the close person receives very little information, even if the patient has given his or her consent. Furthermore, a close person must usually discover on their own or be told by the patient if there have been any deficiencies in the patient's basic care. As a rule, close persons have only the opportunity to meet the patient for a few hours during visiting hours. However, they could play a more significant role in supporting patients and their needs during the care process (eg, in place of the aforementioned care assistant). However, it is first necessary to create clear rules for the close person's role, for example, on how to define the support a close person can provide during treatment, how many close persons are allowed to be present, and whether patient rooms should have a staggered schedule for the close person's support visits. In addition, the patient should clearly name to whom he/she gives consent to act in the role of a close person.

The presence of a close person in outpatient clinics and wards is most often decided from the point of view of whether their presence interferes with the administration of treatment or the performance of a procedure, but not from the perspective of whether it benefits the patient and his or her coping with an illness or its symptoms. If, due to the effects of a disease and its symptoms, a patient does not understand or remember the treatment instructions received, it is difficult for a close person to help the patient due to a lack of standing in the current healthcare system. Close persons must then reach out to the hospital's medical staff and, if that fails, to the patient ombudsman in order to be more fully informed about the patient's case. Disease information, examination results, and treatment guidelines are often requested in these cases through written complaint procedures (5). The result is a care process that arouses uncertainty and feelings of disregard.

How can the support of a close person be rooted in the patient's care process, starting from the acute phase of the disease or severe symptoms, in a professional and organization-oriented care culture that emphasizes the patient's independent coping? A response to this issue can be found in the definition of service as a process (6,7) in which different elements of care are provided, knowledge and information are exchanged, and mutual understanding of care is achieved. The care process is a service process that creates the value of a service in the context of the service provider and the customer with his/her social, cultural, individual, and collective life (7,8). Similarly, the value of a patient's well-being can be seen to be formed in the context of the healthcare professionals treating patients supported by those close to them. If well-being is considered to be a value that the care process is trying to achieve, it is necessary to connect its context of the actors to which a patient's experience of value is formed.

However, the service process includes more than various mechanical stages that progress in terms of time and function. This process emphasizes creating value and *creating value together* (7,8). That is, the patient creates values that are relevant to them, such as physical and mental well-being. Similarly, other actors also create values that can be individual, collective, or social (8). Most often, they are values drawn up in a broader composition, formed in healthcare organizations and municipal or state administrations. As a result, the formation of realized values is complex, as it affects the connection of the care process with other social and healthcare systems.

**Figure 1.**
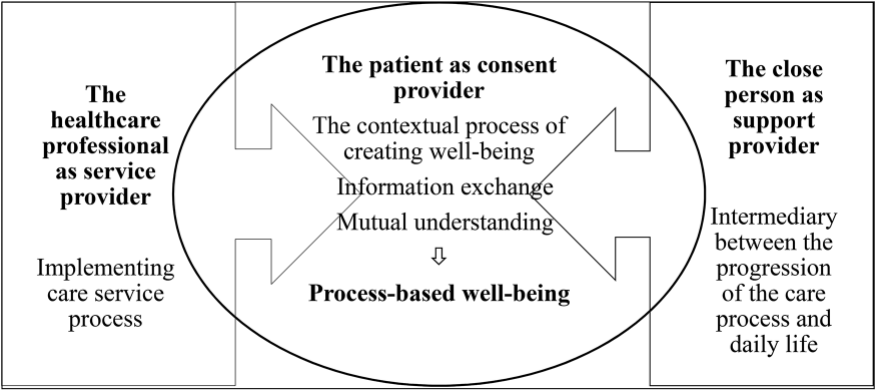
Patient process-based well-being with the support of a close person.

Value creation in the care process is based on the roles of different actors. These roles should be as clear as possible so that none of them dominate the care process (9). Figure 1 shows that healthcare professionals (ie, service providers) play the main role in *providing care* based on their expertise and employment. The patient (ie, consent provider) plays the main role in *consenting to care*, and exercising the rights to self-determination and to information. The close person (ie, the support provider) plays the main role in *supporting the patient's care*, which is realized during the patient's care process in the home and/or at the place of treatment.

The care process can be viewed as the exchange of information and the formation of a mutual understanding based on the need for care. A close person can facilitate this process by mediating between the patient and the doctor. From the perspective of a patient ombudsman, this receiving and providing information and finding a mutual understanding often creates difficulties that lead to complaints being made by close persons (5). Mutual understanding can be connected to the mutual value creation that can occur between a service provider and a patient and/or a close person or all parties together. The concept of different actors collaborating to expand a mutual value is known as co-creation (10). The mutual context of the service process can produce process-based well-being for the patient, for their close persons, and for the healthcare professionals.

### Recommendations

The patient's well-being in the care process can be viewed as comprising process-like service experiences, and perceptions of their own well-being, and the need for support from their close persons when confronting illness. These experiences and perceptions are based on the exchange of information and mutual understanding between the patient (ie, consent provider) with their social context (ie, support provider) and service provider (ie, healthcare professional) in achieving treatment results, in effect creating mutual value for a satisfying care process.

## Conclusion

Strong professionalism and organizational focus in the implementation of care processes undermine customer orientation and its effect on close persons’ support. The patient's services are greatly facilitated when the patient can share their burden with a close person. A close person acts as a supporter of the patient when the patient's resources to respond to the implementation of care and everyday activities are weakened; they compensate for the patient's lost resources.

Customer-centricity has become more prevalent in organizations as values have changed in society. Over time, the values that prevail in society tend to integrate with the values of organizations (10). Currently, we live in a world of desirable values, such as well-being, inclusion, and equality. An organization that wants and is able to monitor the state of the present with a national and global perspective allows values to be reflected in its organizational culture, which embodies the same meaning as the organization itself (10). The support of a close person is the expansion of the mutual value network of patient care, which, in addition to providing the individual and organizational human resource benefits that result from patient process-based well-being, improves the service process. The need for support for patient well-being could thus be a value that welfare states decide to pursue in the near future.

## References

[1] Reher DS. Strong family and low fertility: A paradox? In: Zuanna GD Micheli GA, eds. Family ties in Western Europe. European Studies of Population Vol. 14. Springer; 2004:45-76.

[2] Moor N Komter A. Family ties and depressive mood in Eastern and Western Europe. Demographic Res. 2012;27(8):201-32.

[3] Chan GK Bitton JR Allgeyer RL Elliott D Hudson LR Moulton Burwell P. The impact of COVID-19 on the nursing workforce: A national overview. OJIN. 2021;26(2): 1–16.

[4] World Health Organization. Nursing and midwifery. (Accessed August 31, 2022). https://www.who.int/news-room/fact-sheets/detail/nursing-and-midwifery

[5] HUS Potilasasiamiehet. Potilaan oikeuksien toteutumisen vuosiraportti 2020. [Author’s translation: HUS Patient Ombudsmen. Annual report on the implementation of patients’ rights. 2020. (Accessed August 29, 2022). https://husinvuosi2020.fi/wp-content/uploads/sites/5/2021/06/HUS_Potilaan_oikeuksien-toteutumisen_vuosiraportti_2020.pdf

[6] Osborne SP Radnor Z Nasi G. A new theory for public service management? Toward a (public) service-dominant approach. ARPA. 2012;43(2):135-58.

[7] Grönroos C Voima P. Critical service logic: Making sense of value creation and co-creation. JAMS. 2013;41(2):133-50.

[8] Helkkula A Kelleher C Pihlström M. Characterizing value as an experience: Implications for service researchers and managers. JSR. 2012;15(1):59-75.

[9] Jamil I Askvik S Hossain F. Understanding administrative culture: Some theoretical and methodological remarks. Int J Public Adm. 2013;36(13):900-9.

[10] Ramaswamy V. It’s about human experiences… and beyond, to co-creation. Ind Mark Manag. 2011;40:195-6.

